# Novel CB1 receptor antagonist BAR-1 modifies pancreatic islet function and clinical parameters in prediabetic and diabetic mice

**DOI:** 10.1038/s41387-020-0110-0

**Published:** 2020-03-04

**Authors:** Lesly Nava-Molina, Toyokazu Uchida-Fuentes, Héctor Ramos-Tovar, Martha Fregoso-Padilla, Marco Aurelio Rodríguez-Monroy, Ana V. Vega, Gabriel Navarrete-Vázquez, Erik Andrade-Jorge, Rafael Villalobos-Molina, Ricardo Ortiz-Ortega, Alonso Vilches-Flores

**Affiliations:** 1 Unidad de Biomedicina, FES Iztacala, Universidad Nacional Autónoma de México. Av. de Los Barrios 1, Los Reyes Iztacala, C.P., 54090 Tlalnepantla, Mexico; 2 Facultad de Farmacia, Universidad Autónoma del Estado de Morelos. Av. Universidad 1001, Chamilpa, C.P., 62209 Cuernavaca, Morelos Mexico

**Keywords:** Diagnostics, Dietary carbohydrates, Feeding behaviour

## Abstract

**Backgrouds:**

Cannabinoid receptor antagonists have been suggested as a novel treatment for obesity and diabetes. We have developed a synthetic cannabinoid receptor antagonist denominated BAR-1. As the function and integrity of a β-cell cellular structure are important keys for diabetes onset, we evaluated the effects of pharmacological administration of BAR-1 on prediabetic and diabetic rodents.

**Methods:**

CD-1 mice fed a hypercaloric diet or treated with streptozotocin were treated with 10 mg/kg BAR-1 for 2, 4 or 8 weeks. Body weight, oral glucose tolerance test, HbA1c, triglycerides and insulin in serum were measured. In isolated islets, we evaluated stimulated secretion and mRNA expression, and relative area of islets in fixed pancreases. Docking analysis of BAR-1 was complemented.

**Results:**

BAR-1 treatment slowed down weight gain in prediabetic mice. Fasting glucose–insulin relation also decreased in BAR-1-treated mice and glucose-stimulated insulin secretion was increased in isolated islets, without effects in oral test. Diabetic mice treated with BAR-1 showed a reduced glucose and a partial recovery of islet integrity. Gene expression of insulin and glucagon showed biphasic behaviour, increasing after 4 weeks of BAR-1 administration; however, after 8 weeks, mRNA abundance decreased significantly. Administration of BAR-1 also prevents changes in endocannabinoid element expression observed in prediabetic mice. No changes were detected in other parameters studied, including the histological structure. A preliminary in-silico study suggests a close interaction with CB1 receptor.

**Conclusions:**

BAR-1 induces improvement of islet function, isolated from both prediabetic and diabetic mice. Effects of BAR-1 suggest a possible interaction with other cannabinoid receptors.

## Introduction

In recent years, several evidences have enligthened the active role of the endocannabinoid system (ECS) in food intake and metabolism regulation, as ECS overactivity is strongly associated with the onset of obesity, type 2 diabetes, and some risk factors of metabolic syndrome like impaired insulin sensitivity, glucose intolerance, and dyslipidaemia^1–8^. In the past decade, cannabinoid receptor antagonist rimonabant was studied as novel pharmacological option for obesity and type 2 diabetes treatment. Rimonabant blocks cannabinoid receptor 1 (CB1r) and regulates the activity of the endocannaboid system in different organs. Clinical and experimental reports indicated beneficial effects in food intake, body weight, adiponectin and leptin levels, insulin resistance, glucose and lipids homeostasis^9–13^. However, it was withdrawn for safety reasons after neurological side effects associated with severe depression were reported. Nevertheless, rimonabant studies opened a new research line dedicated to elucidate the functions of the ECS and to find alternative compounds to regulate the ECS^14,15^, such as BAR-1 (1-(4-chlorophenyl)-2-(2,4-dichlorophenyl)-*N*-(1-piperidinyl)-1*H*-benzimidazole-5-carboxamide).

In pancreatic islets, the function of ECS has been related with glucose-regulated hormone secretions and the development of dysfunction of this endocrine tissue. All the elements of the ECS are present in rodent and human islets, but there is not a consensus regarding if the activation of CB1 and CB2 receptors results in stimulation^16–27^, or inhibition of insulin secretion^28–35^. Some studies indicate that cannabinoid agonists and antagonists have either acute or chronic effect increasing islets function, in models of diet-induced obesity and diabetes^23,27,36,37^, at high glucose concentrations^2,30,31,38,39^, or after an overactivation of ECS^20–22,24^. In human islets, CB1r- and CB2r-selective antagonists increase insulin secretion, but the effects of agonists have been controversial^22,30,32^. In islets isolated from rats with high basal levels of insulin secretion, rimonabant ameliorate its dysfunction^25^. In prediabetic animal models, administration of CB1r antagonists rimonabant and Ibipinabant induces significant improvements in insulin secretion, glucose tolerance and islet morphology^37,40,41^.

Previously, we reported the acute effects of a new synthetic analogue of rimonabant, denominated BAR-1, in isolated pancreatic islets from rat^20^. This compound has a benzene group incorporated to rimonabant molecule that changes CB1 receptor affinity and possibly its activity. At 1 μM concentration, acute exposure to BAR-1 modified mRNA abundance of CB1r, glucagon, PDX-1 and glucokinase in isolated islets in response to changes in glucose concentration in media. Glucose-stimulated insulin secretion was enhanced in the presence of BAR-1 and partially reduced anandamide effects. In continuation with our previous reports that focus on the ECS function in pancreatic islets^21,22^, considering the useful and easy model of prediabetes induced in mice with high-fat diet^36^, and the strong evidences of the relationship between pancreatic islet physiology and diabetes, the aim of the current study is to evaluate the effects of pharmacological administration of BAR-1 in pancreatic islets from mice with streptozotocin (STZ)-induced diabetes and in a prediabetic model induced with hypercaloric diet (HCD); we described the effects of BAR-1 with particular interest on its influence at gene expression, insulin secretion and the morphology of pancreatic islets.

## Materials and methods

### Reactives

BAR-1 was synthetized by Navarrete-Vázquez and colleagues^20^, and its synthesis has been described previously. Dulbecco’s modified Eagle’s medium (DMEM) low glucose, Fetal bovine serum (FBS), penicillin/streptomycin, l-glutamine, collagenase type V, Histopaque 1077, STZ and PCR primers for CB1r, CB2r, MAGL, NAPE-PLD, FAAH, DAGL and 18s rRNA were purchased from Sigma Aldrich (St. Louis MO, USA). Preproinsulin (PPI) and preproglucagon (PPG) were obtained from Qiagen (West Sussex, UK). Real-time PCR master mix and reagents were purchased from Fermentas/Thermo Scientific (Madison, WI, USA) and Invitrogen (Grand Island, NY, USA). A1cNow kit was from ChekDiagnostics (Diagnodistributions, USA), and insulin and glucagon enzyme-linked immunosorbent assay (ELISA) kits were obtained from Alpco (Salem, NH, USA). Anti-insulin primary antibody and secondary antibodies were obtained from Santa Cruz Biotechnology.

### Animal models

Male CD-1 mice (4 weeks old) were obtained from the local animal facility and maintained under controlled conditions according to official Federal Guidelines NOM-062-ZOO-1999. All experiments were approved by the institutional ethics committee of the Facultad de Estudios Superiores de Iztacala, Universidad Nacional Autonoma de Mexico. Experiments are also in accordance with the recommendations in the Guide for the Care and Use of Laboratory Animals of the National Institutes of Health. Experimental diabetes was induced with a single dose of STZ, 120 mg/kg dissolved in citrate buffer pH 4.0. Glycaemia was measured 72 h later; only mice with fasting glycaemia over 200 mg/dl were selected for the study. Prediabetes was induced with a HCD that have 30% extra fat, compared with normal diet and ad libitum intake of a 20% sucrose solution instead of water. Control groups were fed with standard rodent chow food: Kcal% carbohydrates 60, fat 12, 3.9–4.1 kcal/g; HDC groups had partial Kcal% carbohydrates 45, fat 40, 4.9–5.1 kcal/gm. Mice were treated with BAR-1 administrated orally at a 10 mg/kg dose, or vehicle solution with dimethyl sulfoxide 20%. Diabetic mice received BAR-1 daily for 2 weeks, whereas prediabetic mice received treatment for the 4 or 8 weeks. Each group contained *n* = 8 animals, selected randomly.

### Oral glucose tolerance test and clinical parameters

Oral glucose tolerance test (OGTT) was performed after administration of a dose of glucose 2 g/kg. Previously, animals were fasted for a 16 h period. Blood samples were obtained from the tail vein at 0, 30, 60, 90 and 120 min, and glucose levels were measured with One Touch UltraMini Glucometer (Johnnson&Johnnson). Body weight was registered every week in all groups and triglycerides in the blood were detected 1 day before OGTT with Accutrend GCT equipment (Roche Diagnostics). In diabetic mice, glycosylated haemoglobin (HbA1C) was determined with an A1CNow+ (L1423226) portable device. For prediabetic animals, body size was measured after 4 and 8 weeks of treatment and fasting glucose–insulin ratio was determined. Serum insulin content was measured by ELISA.

### Histological analysis

Pancreases from three mice of each group—control, BAR-1-treated and STZ-treated animals—were fixed with Bouin’s solution, paraffin-embedded and cut in 7 μm sections; *n* = 8 sections of three pancreas per condition. Islet contrast from acinar tissue was obtained with haematoxylin–eosin standard stain, and specific islets area was determined in 20,000 μm^2^ of pancreas tissue, using a Microscope Digital Eyepiece MDE-130 coupled to ScopeImage 9.0 software. Immunodetection was conducted using a rabbit polyclonal anti-insulin antibody (Santa Cruz Biotechnology H-86 sc-9168) or mouse monoclonal anti-glucagon (Sigma G2654 clone K79bB10), and secondary antibodies anti-rabbit IgG Alexa 488 and anti-mouse IgG Alexa 549 (Invitrogen A11008, 571716 and A11030, 134546), used under the same conditions as previously reported^20^. Nuclear DNA was detected with Hoescht (Vectorlabs). Images and intensity signal were obtained in a Leica TCS SP2 inverted confocal laser scanning microscope (Leica, Leidemberg, Germany).

### Islet isolation for static insulin and glucagon secretion, and gene expression analysis

Islets from five prediabetic and control mice, treated with BAR-1 or vehicle for 4 and 8 weeks, were isolated by collagenase digestion and Histopaque 1077 density gradient. A day before, OGTT was conducted. Hand-picked, size-matched islets were maintained overnight in DMEM medium with 5.5 mM glucose, 10% FBS, 2 mM l-glutamine and antibiotics. Static insulin secretion was evaluated with batches of five islets pre-incubated 1 h in physiological buffer^21^, containing 5 mM glucose at 37 °C. Then, islets were treated with 16 mM glucose for 1 h and insulin release was determined by ELISA, according to the manufacturer’s instructions. In a similar procedure, static glucagon secretion was determined in five islets pre-incubated 1 h at 37 °C in physiological buffer with 16 mM glucose and then treated with 10 mM arginine for 1 h. Hormone content in media was also quantified by ELISA. For gene expression analysis, RNA was collected with TRIzol reagent and isopropanol, and quantified by spectrometer. Synthesis of cDNA by reverse-transcription reaction was performed with 0.5 μg of total RNA and real-time PCR with 20 ng/μl of cDNA. Primers and amplification conditions have been reported previously^20–22^. Relative expression of mRNAs was determined after normalization against 18s rRNA, as the internal reference gene, and was calculated by the 2^−ΔΔ^Ct method^42^.

### In-silico analysis of rimonabant interaction with CB1r and prediction of BAR-1

Ligands were optimized by modelling with GaussView 6.0.16, considering the protonation status under physiological conditions (pH 7.4). The optimization of the molecules was performed with Gaussian 16^43^ and a semi-empirical method (AM1). Crystal structure of the Human CB1 receptor (PDB code: 5TGZ^44^) was taken from the Protein Data Bank (PDB). Docking parameters for ligands (rotational bonds, degrees of torsional freedom, atomic partial charges and non-polar hydrogen bonds) and binding parameters were assigned with AutoDock Tools 1.5.6^45^ and Raccoon^46^, employing a hybrid Lamarckian genetic algorithm^47^ to obtain a population of 100 randomly placed conformers per generation. Kollmann’s partial charges were assigned to all proteins. Grid parameters were established by using a 70 × 60 × 60 Å grid box, coordinates of *X* = 42.474, *Y* = 28.652 and *Z* = 319.997, and a 0.375 Å mesh separation. The calculations were executed on AutoDock4 with a Linux operating system (Fedora 22). Finally, the lowest energy state, expressed as Gibbs free energy (Δ*G*), was obtained for each compound. The dissociation constant (*K*_d_), −log dissociation constant (p*K*_d_), number of interactions, and distance and type of binding were determined with the Visual Molecular Dynamics programme (VMD v.1.8.6)^48^ and Discovery studio 4.0 client.

### Statistical data analysis

Data are expressed as means ± SEM obtained from six to eight mice per group, or from four individual in-vitro experiments. One-way analysis of variance with Bonferroni’s post-hoc test were used for analyses; differences between treatments were considered statistically significant at *p* < 0.05.

## Results

### Effects of BAR-1 on metabolic and clinical parameters

**Fig. 1:**
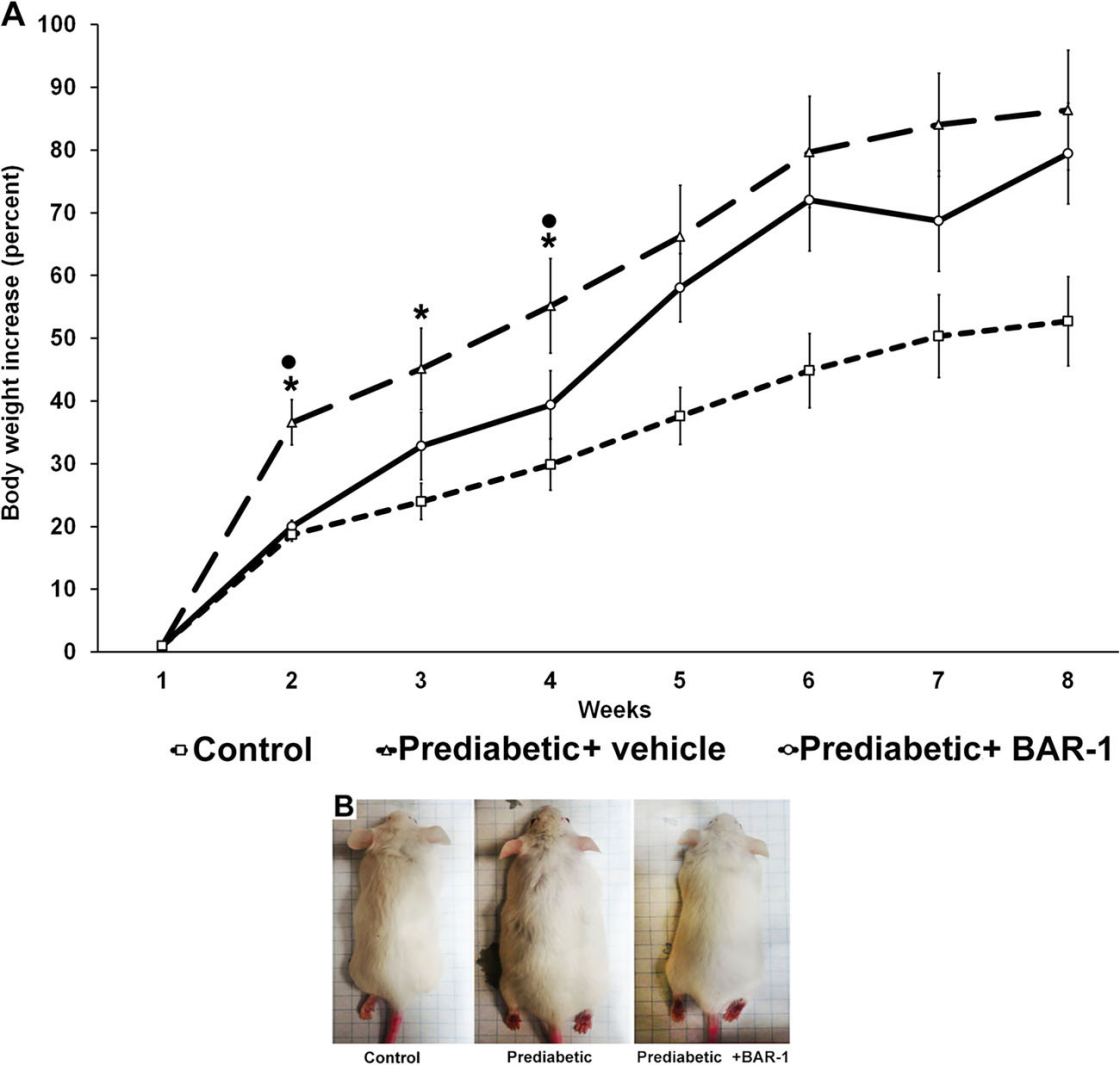
BAR-1 effect in body weight. **a** Effect of BAR-1 treatment (10 mg/kg) for 8 weeks in the body weight of prediabetic and control mice. Data are normalized to their respective initial weight at week 1; means + SEM, each group with *n* = 8 mice. *p < 0.05 vs. control, black dot vs. vehicle treated. **b** Comparative sizes of a representative mouse from control, prediabetic and prediabetic BAR-1-treated groups, at 8 weeks.

Administration of BAR-1 at a 10 mg/kg dose prevented weight gain in prediabetic mice fed with HCD at least for the first 4 weeks (Fig. 1a). However, after week 5, all mice on HCD gained weight to the same extent without regarding the exposure to BAR-1. Size and body mass index showed a similar behaviour (Fig. 1b). Nevertheless, animals treated with BAR-1 seemed to have less abdominal adipose mass.

**Fig. 2:**
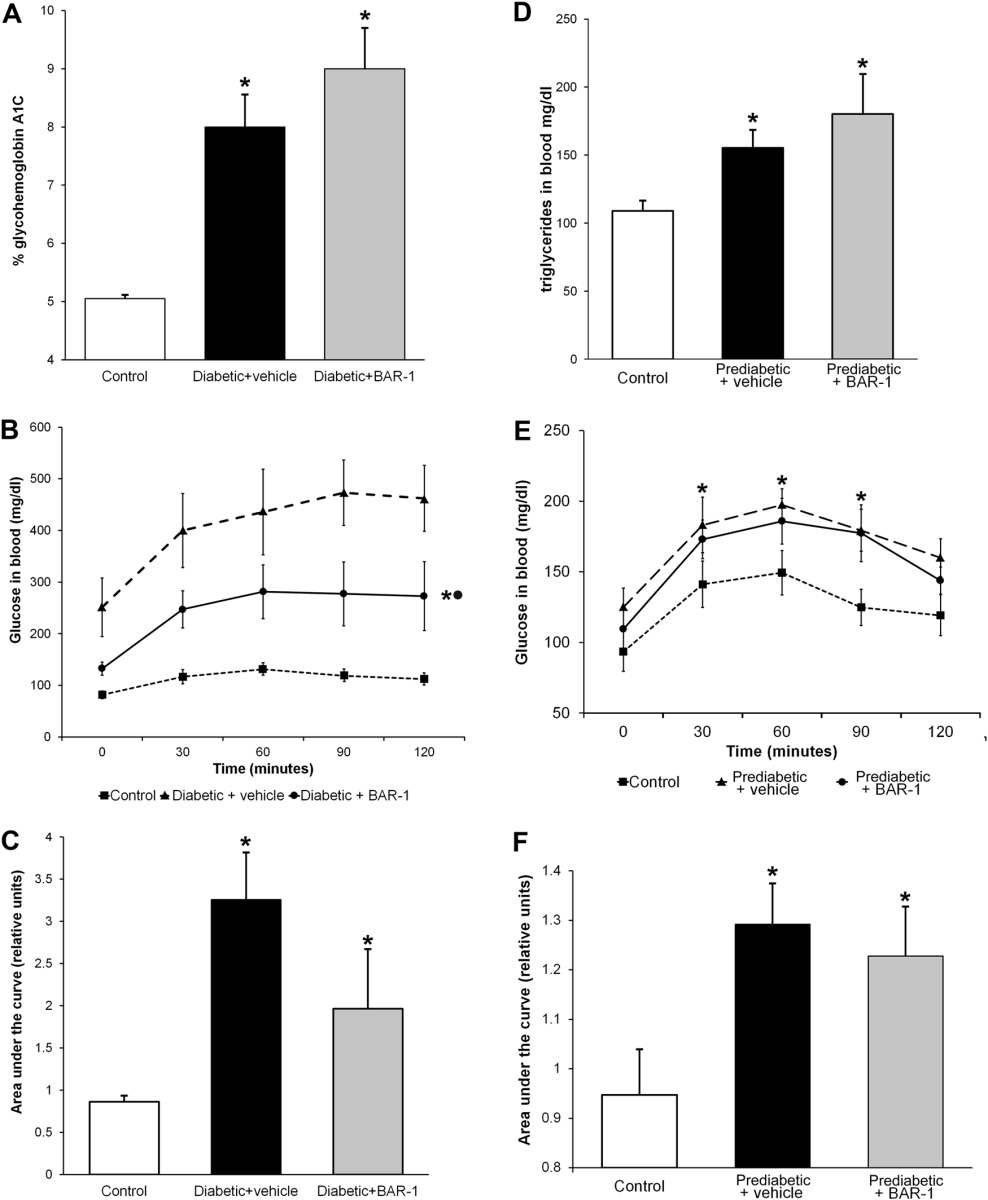
BAR-1 effects on glycated HbA1C, oral glucose tolerance test and triglyceride. Effects of BAR-1 treatment (10 mg/kg oral) in glycated HbA1C (**a**) and OGTT (**b**) of diabetic mice after 3 weeks and triglyceride blood levels (**d**) and OGTT (**e**) of mice fed with high caloric diet (prediabetic) after 8 weeks. Area under the curve was analysed for each group (**c**, **f**). Data are presented as means + SEM, *n* = 8 mice per group. *p < 0.05 vs. control, black dot vs. vehicle treated.

In diabetic groups, weight, size and body index were unaffected by BAR-1. Likewise, treatment did not modify HbA1c glycated percent (Fig. 2a). However, OGTT presented a significant reduction with respect to the vehicle-treated group (Fig. 2b), although the area under the curve is still augmented in comparison with that of control mice (Fig. 2c). Prediabetic mice with or without BAR-1 treatment showed an increased triglyceride levels in blood (Fig. 2d) and a significant increase in OGTT and the area under the curve, when measured after 8 weeks (Fig. 2e, f). Insulin resistance index, considering basal glucose and insulin in serum, showed a progressive increase in prediabetic (11.5 ± 4.6) and BAR-1-treated mice (10.4 ± 4.6) compared with control mice (8.1 ± 2.1).

### BAR-1 treatment improved pancreatic histological structure in diabetic model, but not in prediabetic model

**Fig. 3:**
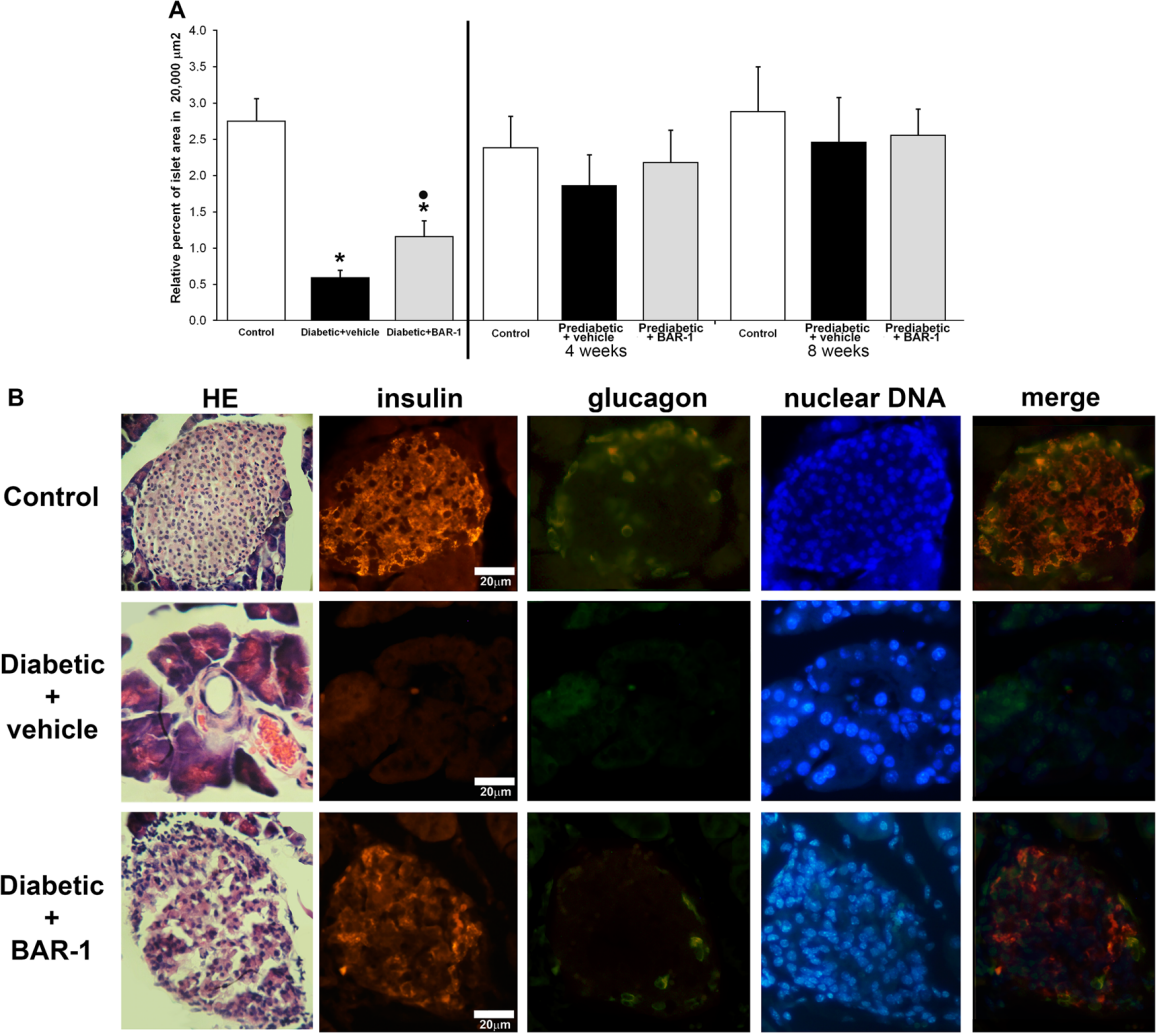
Histological effects of BAR-1 in pancreatic islets. **a** Analysis of islet relative area, in pancreas sections from prediabetic mice of 4 and 8 weeks, or with induced diabetes, treated or not with BAR-1 (10 mg/kg). Data are presented as means + SEM, *n* = 8 sections of three pancreas per condition. *p < 0.05 vs. control, black dot vs. vehicle treated. **b** Islet identification by HE and immunohistochemical determination of cells positive to insulin (CY5), glucagon (FITC) and nuclei (DAPI) in pancreas sections from control and diabetic mice treated with vehicle or BAR-1. Images are representative of five samples per group.

We analysed the histological structure of pancreas from control, diabetic and prediabetic mice, treated with BAR-1 for 2 (diabetic mice) or 4 and 8 weeks (prediabetic mice). As observed before, STZ-induced diabetic animals showed a significant decrease in islet areas. Interestingly, a partial recovery on islet area was observed after BAR-1 treatment. In Fig. 3a we show a representative image of similar observations made on three different animals; all of them show a partial recovery or perhaps preservation of islet morphology. More interestingly, after 2 weeks of BAR-1 treatment, diabetic mice islets showed positive insulin immunoreactivity, similar in intensity to control pancreas; however, in contrast with control pancreas, glucagon signal was more intense and it showed some overlap with insulin immunoreactivity (Fig. 3b). Nevertheless, this finding supports the notion that the ECS is more relevant to preserve the islet integrity than thought before. Regarding the prediabetic model, as expected, islet areas in different sections of the pancreas did not show significant changes between control and prediabetic animal, although there was a tendency to have more islet areas in the last group. Neither group was affected by BAR-1.

### BAR-1 modifies insulin mRNA and secretion in islets isolated from prediabetic mice

**Fig. 4:**
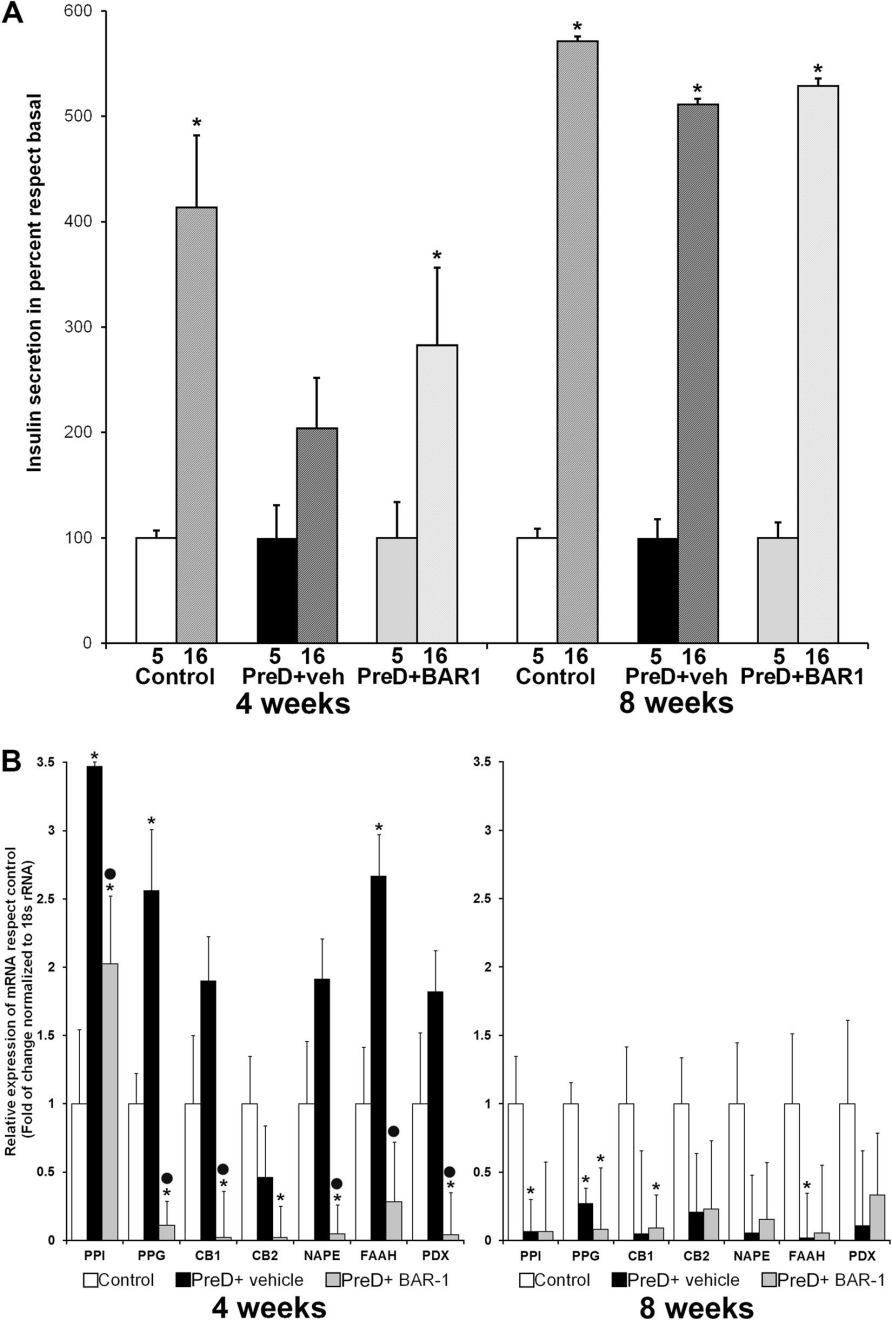
Glucose-induced insulin secretion and mRNA relative expression. Glucose-induced insulin secretion (**a**) and mRNA relative expression (**b**) in isolated islets from control and prediabetic mice of 4 and 8 weeks, with or without BAR-1 treatment. Insulin secretion was calculated in percent regarding basal 5 mM glucose for each group. mRNA relative abundance is expressed in fold of change regarding control islets. Data are presented as mean + SEM, *n* = 5 islets per condition for hormone secretion and *n* = 50 for mRNA expression, all determinations were performed in triplicate. *p < 0.05 vs. control, black dot vs. vehicle treated.

Previously, we had reported that acute exposure (1 h) to BAR-1 was enough to induce an increase of PPI mRNA expression and glucose-induced insulin release in isolated rat islets^20^. Therefore, we set to study glucose-dependent hormone release and the expression levels of several genes relevant to the islet functional properties and survival. Isolated islets recovered at 4 weeks from prediabetic mice presented a decrease in glucose-stimulated insulin secretion and this effect was prevented with BAR-1 treatment (Fig. 4a). However, after 8 weeks, no evident changes in glucose-stimulated secretion were observed between groups. In contrast, glucagon secretion (pg/islet/h) remained unchanged at both times and was insensitive to diet or treatment: basal control 14.5 ± 0.2, stimulated 35.6 ± 0.6; basal BAR-1 treated 14.4 ± 0.8, stimulated 36.0 ± 0.3. mRNA expression of PPI, measured by quantitative reverse-transcription PCR (Fig. 4b), increased over three times in islets from prediabetic mice after 4 weeks and it was partially reduced by BAR-1 treatment; however, at 8 weeks, PPI mRNA expression was reduced by ~2-fold compared with the control mice, but it was not reduced further by BAR-1 treatment. A similar behaviour was observed with PPG mRNA; it increased at 4 weeks of HCD diet, but showed a reduction at 8 weeks; in this case, BAR-1 treatment decreased PPG expression to similar levels at both times studied.

In our previous report with isolated islets treated with BAR-1^20^, significant changes in CB1 and CB2 receptor expression were observed. Therefore, for this study we analysed in islets from prediabetic mice the effect of oral BAR-1 treatment on mRNAs relative abundance. We found that CB1 showed a similar expression pattern to PPI y PPG, increasing at week 4 and decreasing at week 8. In contrast, CB2 mRNA levels seem to be unaffected by either the diet or the treatment with BAR-1.

The changes in mRNA due to time of HCD intake were not only observed in hormones mRNA and cannabinoid receptors, but also the enzymes involved in endocannabinoids synthesis were affected in a similar manner. mRNAs for NAPE-PDL and FAAH enzymes show a biphasic behaviour dependent on the exposure to HCD in prediabetic mice. Furthermore, their expression levels were reduced by treatment with BAR-1. The changes in mRNA relative expression of PPI, PPG, CB1, FAAH and NAPE may be related to changes on the transcription factor PDX-1. As PDX-1 has been identified as a master transcription factor, essential to proper islet function, changes on its mRNA and expression may be mirrored by genes relevant to islet physiological functions. As it is shown in Fig. 4b, both diet and treatment with BAR-1 influence this master gene in close resemblance to what happened with PPI and PPG.

### In-silico analysis suggests a similar effect of BAR-1 and rimonabant

**Fig. 5:**
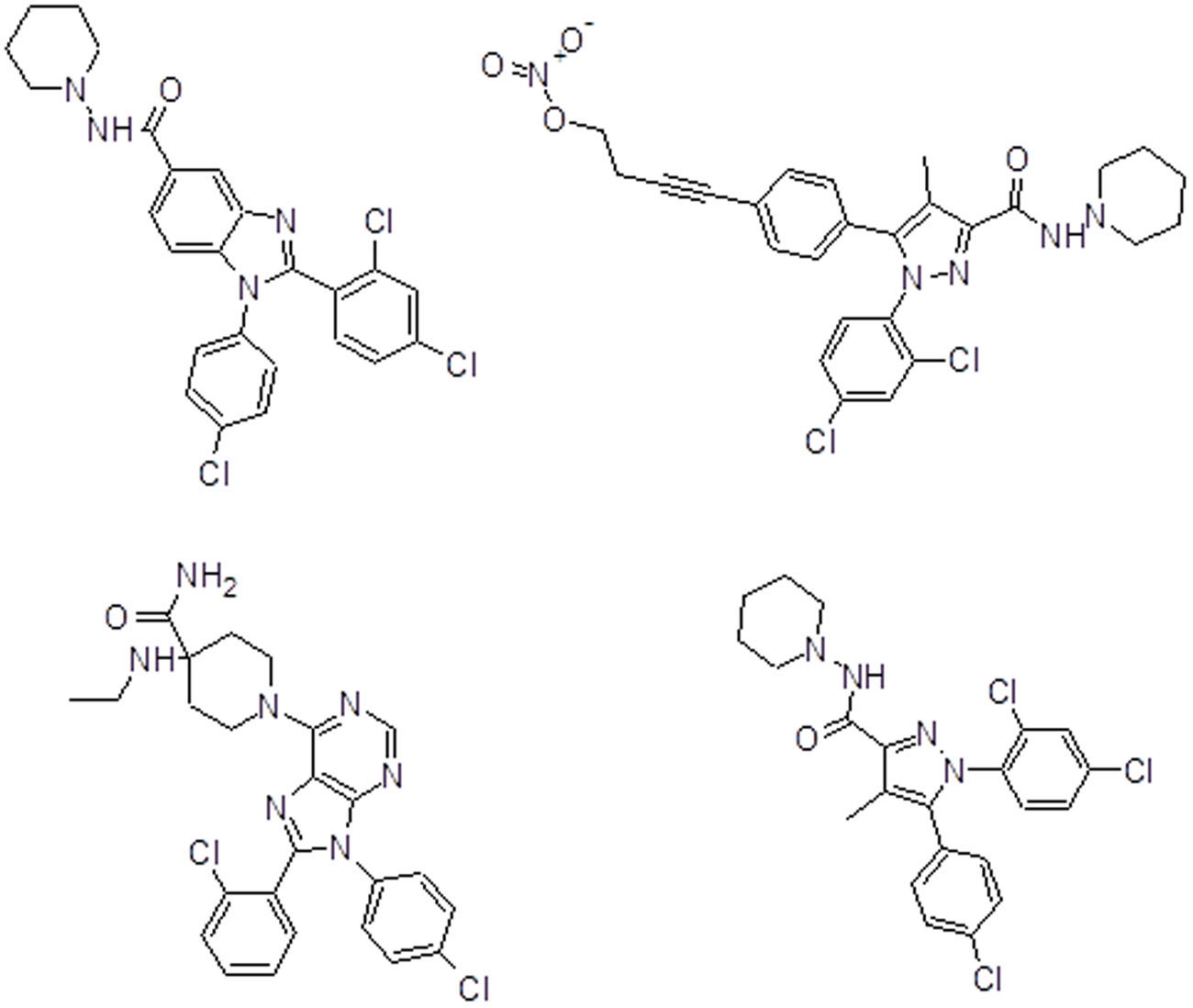
Chemical structure and analysis of BAR-1. Chemical structures of BAR-1 (**a**), AM6538 (**b**), rimonabant (**c**) and otenabant (**d**).

BAR-1 molecular structure (Fig. 5) and three reference molecules (AM6538, rimonabant and otenabant) were modelled with a semi-empirical method (AM1) and then docked with the human CB1r, obtaining ΔG, *K*_d_ and p*K*_d_ for the interactions between human CB1r and the ligands (Supplementary Table 1). Molecular docking results show a high affinity of BAR-11 similar to the reference molecules and even more than some references.

The main amino acid residues involved in ligand-CB1 recognition for BAR-1 and some reference drugs were identified (Supplementary Table 2). Some amino acid residues are the same for the BAR-1 and the reference drugs such as Val196, Trp356, phe174, His178, Phe170, Ser383 and Cys386, and also the kind of interaction are shown (Supplementary Table 3). These amino acid residues have been described as important for the antagonism of the human CB1r^44^.

The binding mode and main amino acid residue interactions with CB1r turned out to be very similar for BAR-1 and the reference molecules (Supplementary Fig. A, B). Moreover, BAR-1 and the reference drugs AM6538, rimonabant and otenabant exhibited the same pattern in relation to the binding mode (Supplemental Figs. A, B). The validation of the molecular docking was performed by comparison of docked and crystallographic AM6538, obtaining the same pattern of interaction with the receptor for both molecules (Supplementary Figs. B–D and D’).

## Discussion

In a previous work, we reported for the first time the potential use of BAR-1, a novel synthetic analogue of rimonabant^20^. Acute treatment with BAR-1 in pancreatic islets isolated from rats demonstrated significant effects on gene expression, glucose-stimulated insulin secretion and partial anandamide antagonism, conducting to the next in-vivo experiments performed in animal models of prediabetes and diabetes states. Similar studies with cannabis derivatives^27,38,49^, novel CB receptors antagonists and agonists^39,40^, have been performed in different animal models, providing evidences for an important role of the ECS in pancreatic islets function and the development of therapeutic alternatives for control of obesity and diabetes.

Prediabetic and diabetic mice presented some short-term improvements after BAR-1 treatment, preventing body weight gain during the first 4 weeks; despite feeding HCD, the reduction glucose-stimulated insulin secretion was partially reverted and the expression of genes key to islet function was stimulated. These effects are consistent with previous observations with rimonabant-treated Zucker fatty or diabetic rats and diabetic OLETF rats^25,37,39–41^. However, continuous development of metabolic damage in these models finally overturn these changes produced by the CB1r blockade. In this study, we focused our effort on studying the pancreatic islets function in prediabetic mice and we think that the changes we observed on isolated islets account, at least partially, for the slowdown of the weight-gain curve observed within the first 4 week of exposure to HCD. However, considering the presence and activity in other organs, including the nervous system, adipose tissue and liver^5,50^, we do not rule out the possible indirect or direct influence in pancreatic islets.

Another explanation for the brief benefit of BAR-1 treatment could be that long-term exposure induced adaptation, so that modified diets initially increase the production of endocannabinoids and enhance islet dysfunction, but in the long-term its levels become normal again^30,31,36,41^. Previous studies have demonstrated that the expression of receptors and enzymes of the ECS in rat, mouse and human islets can be adaptable under chronic exposure to glucose and CB1r or CB2r agonists^2,16,17,21,22^. Mice treated with BAR-1 presented a significant reduction in PPG and CB1r mRNAs expression at 4 and 8 weeks, suggesting an interesting regulatory role of the ECS in alfa-cells, as previous observations indicate^21,22,35^. Although we did not observe changes in glucagon secretion after BAR-1 administration, other studies have associated alfa-cells activity with the function of CB1r^21,22,24^. The downregulation of PDX-1 expression is very interesting, because many functions and characteristics specific of pancreatic islets are determined by this transcription factor. In prediabetic mice, PDX-1 mRNA expression seem to go under an adaptive processes between weeks 4 and 8, but CB1r blockade with BAR-1 treatment prevented partially its downregulation. Therefore, we think that ECS activation plays a role on diabetes developing and it is involved with small changes in islets during this process.

In diabetic mice, BAR-1 treatment reduced OGTT significantly, but performance of specific experiments with isolated islets from diabetic mice implies more technical specifications and the possibility to obtain an insufficient number; therefore, we decided to analyse histological changes in whole pancreas. To our surprise, the presence of insulin-positive cells in mice with BAR-1 treatment was considerably higher than in untreated diabetic subjects, suggesting a protective effect. This is in good agreement with observations of Kim et al.^30^ regarding CB1r blockage with AM251 also led to increases in β-cell area. Controversial findings regarding activation and blockage of CB1r have been reported; direct and indirect effects in β-cells survival and function have been shown, including morphological changes in islets and modulation of cellular pathways that promotes apoptosis^30,31,33,40^. These discrepant observations may be due partially from different animal models and alternative treatments as follows: pharmacological agent, dose, via and time. For future studies, it could be interesting to test different options of administration of BAR-1, in other animal models, and possible effects in other organs involved in metabolism and with ESC activity previously and widely reported, such as the gut, adipose tissue, liver, muscle and brain^4,27,37,50^.

Undesirable secondary effects of rimonabant treatment during clinical trials, with diabetic or metabolic syndrome patients, gave a reason to wish for safer alternative analogues^10–12^. Comprehensive studies describing binding of rimonabant to the major binding pocket of human CB1r^51^ give us reasons to think that BAR-1 binds to the same cannabinoid receptor. The main kind of interaction for all tested molecules in this study—BAR-1, AM6538, rimonabant and otenabant—is hydrophobic. The addition of an extra aromatic ring in BAR-1 increases the hydrophobic interactios and, therefore, increasing the affinity for the human CB1 receptor, estimated by Δ*G*, *K*_d_ and p*K*_d_. Because of its similarities, BAR-1 is thought to be an antagonist of CB1r; however, in view of the complexity of the responses observed, we cannot rule out a possible interaction with other cannabinoid receptors such as CB2r or GRP55 as either agonist or antagonist effects. In this first in-vivo approaching, we do not report evidences of secondary effects or predict them, as we used the same 10 mg/kg dose to mimic rimonabant results previously reported^25,40,41^. In forthcoming studies with BAR-1, we will explore different doses and administration protocols, focusing on side effects and the possible interactions with CB1 receptors in brain. Another possibility of BAR-1 interaction could be related to the activity of CB2 receptors and GPR55, requesting in future more extensive pharmacological evaluations.

In conclusion, rimonabant synthetic analogue BAR-1 provided a short-lasting but promising improvement in treatment of prediabetes and diabetes. The strong molecular interaction of BAR-1 with CB1r and its effects on islets morphology and function suggest its potential use as a novel pharmacological agent through modulation of ECS in metabolic alterations.

## Supplementary information

Supplemental material - Legends

Suplemental material - Tables 1, 2 and 3

Suplemental material - Figure A

Suplemental material - Figure B
