# Supplementary material for: Novel CB1 receptor antagonist BAR-1 modifies pancreatic islet function and clinical parameters in prediabetic and diabetic mice
Source: Nutr Diabetes. 2020 Mar 4;10:7. doi: 10.1038/s41387-020-0110-0 (PMC7055595; doi:10.1038/s41387-020-0110-0)
Supplement: Supplementary file 1 — Supplemental material - Legends [file 41387_2020_110_MOESM1_ESM.docx]

**Supplemental material**

**Table 1.** Gibbs free energy (ΔG), the dissociation constant (K_d_), and –log_10_ dissociation constant (pK*_d_*) for the interaction of the test and references molecules with the human CB1r.

**Table 2**. Amino acid residues involved in the interaction between human CB1r and the ligands: BAR-1 and reference molecules.

**Table 3**. Amino acid residues, type of interactions and binding distances in the approach of BAR-1 and references molecules to the human CB1r.

**Figure A**. Binding mode and main amino acid residues in the active site of the human CB1r interacting with the reference drugs and tested molecule (observed by docking simulation): **A**) binding site of BAR-1; **A’**) amino acid residues interacting with BAR-1; **B**) binding site AM6538; **B’**) amino acid interacting AM6538; **C**) binding site otenabant; **C’**) amino acid interacting otenabant; **D**) binding site rimonabant; **D’**) amino acid interacting rimonabant.

**Figure B**. Binding mode of the ligand in the active site of the human CB1r established by molecular docking: **A**) the binding mode of BAR-1 was similar to that of AM6538 (yellow); **B**) a similar binding mode was found for BAR-1 and AM6538 (fuchsia); **C**) a similar binding mode was observed for BAR-1 and Otenabant (ochre); **D**) reproduction of the binding mode of AM6538 obtained by molecular docking (yellow) and by crystal structure (fuchsia; PDB code 5TGZ); **D’**) amino acid residues interaction of AM6538 obtained by molecular docking (yellow) and by crystal structure (fuchsia; PDB code 5TGZ).
