# Supplementary material for: Novel CB1 receptor antagonist BAR-1 modifies pancreatic islet function and clinical parameters in prediabetic and diabetic mice
Source: Nutr Diabetes. 2020 Mar 4;10:7. doi: 10.1038/s41387-020-0110-0 (PMC7055595; doi:10.1038/s41387-020-0110-0)
Supplement: Supplementary file 2 — Suplemental material - Tables 1, 2 and 3 [file 41387_2020_110_MOESM2_ESM.docx]

**Table 1.**

|  | AChE (*Electrophorus electricus*) | | |
| --- | --- | --- | --- |
| ID | ΔG (kcal/mol) | K*_d_* (µM) | pK*_d_* |
| Bar1 | -12.17 | 0.0012 | 8.92 |
| AM6538 | -13.58 | 0.00011 | 9.95 |
| Rimonabant | -11.99 | 0.0016 | 8.79 |
| Otenabant | -11.65 | 0.0028 | 8.54 |

**Table 2.**

| Ligand | Residues |
| --- | --- |
| BAR-1 | Ile119, Ser123, Phe174, His178, Phe170, Ile105, Ser390, Ser383, Cys386, Phe102, Leu193, Ser199, Val196, Trp356, Gly166 |
| AM6538 | Ile119, Ser123, Phe174, Phe170, Phe102, Gly166, Met384, Ser383, Leu387, Cys386, Phe268, Leu193, Val196, Thr197, Trp356, Leu359, Phe200, Trp279, Met363, Leu360 |
| rimonabant | Phe174, His178, Met103, Phe102, Gly166, Val196, Leu193, Ser383, Cys386, Phe379, Leu359, Trp356, Phe170 |
| otenabant | Ser123, His178, Phe174, Met103, Phe170, Phe102, Ile105, Met384, Leu387, Ser383, Phe379, Trp356, Val196, Leu193, Cys386 |

**Table 3.**

| Ligand | Interactions | |
| --- | --- | --- |
| BAR-1 | **hydrophobic interactions:**  Ile119:C----C:Bar1 to 5.30 Å  His178:C----C:Bar1 to 4.69 Å  Phe174:C----C:Bar1 to 5.03 Å  Met103:C----C:Bar1 to 4.85 Å  Phe268:Cl----C:Bar1 to 4.98 Å  Leu193:C----C:Bar1 to 4.95 Å  Val196:C----C:Bar1 to 4.54 Å  Trp356:C----C:Bar1 to 5.45 Å  Gly166:C----C:Bar1 to 3.37 Å  Phe170:C----C:Bar1 to 4.95 Å  Val196:C----C:Bar1 to 4.07 Å | **π-π interaction:**  Phe268:C----C:Bar1 to 5.92 Å  **π-donor interaction:**  Ser383:C----C:Bar1 to 3.06 Å  **π-sulfur interactions:**  Cys386:S----C:Bar1 to 5.51 Å |
| AM6538 | **hydrophobic interactions:**  Phe174:C----C:AM6538 to 5.37 Å  Met384:C----C:AM6538 to 5.03 Å  Met103:C----C:AM6538 to 4.91 Å  Met103:Cl----C:AM6538 to 5.01 Å  Phe102:C----Cl:AM6538 to 4.89 Å  Phe170:C----Cl:AM6538 to 5.04 Å  Val196:C----Cl:AM6538 to 4.36 Å  Leu387:C----Cl:AM6538 to 5.04 Å  Leu387:C----C:AM6538 to 5.02 Å  Cys386:C----C:AM6538 to 5.57 Å  Phe379:C----C:AM6538 to 4.75 Å  Val196:C----C:AM6538 to 3.90 Å  Leu359:C----C:AM6538 to 5.30 Å | **π-donor interaction:**  Ser383:H----C:AM6538 to 3.77 Å  **π-anion interaction:**  Phe200:C----O:AM6538 to 3.38 Å  **Hydrogen bond:**  Trp279:H----O:AM6538 to 2.87 Å |
| otenabant | **hydrophobic interaction:**  Ala380:C----C:Otenabant to 3.75 Å  Ile105:C----C:Otenabant to 4.98 Å  Met103:C----C:Otenabant to 5.18 Å  Met103:C----Cl:Otenabant to 4.70 Å  Phe102:C----Cl:Otenabant to 4.53 Å  Phe170:C----Cl:Otenabant to 5.20 Å  Leu387:C----C:Otenabant to 5.01 Å  Cys386:C----C:Otenabant to 5.40 Å  Val196:C----Cl:Otenabant to 4.48 Å  Val196:C----C:Otenabant to 4.84 Å  Val196:C----Cl:Otenabant to 4.19 Å  Leu193:C----Cl:Otenabant to 5.01 Å  Trp356:C----Cl:Otenabant to 5.50 Å  Leu359:C----C:Otenabant to 5.49 Å | **Hydrogen bond:**  His178:N----H:Otenabant to 3.08 Å  Ser123:O----H:Otenabant to 2.75 Å  **π-π interaction:**  Phe170:C----C:Otenabant to 4.98 Å  **π-donor interaction:**  Ser383:O----C:Otenabant to 3.56 Å  Ser383:O----C:Otenabant to 3.28 Å  **π-sulfur interaction:**  Cys386:S----C:Otenabant to 5.76 Å |
| rimonabant | **hydrophobic interaction:**  Phe174:C----C:Rimonabant to 5.06 Å  His178:C----C:Rimonabant to 4.45 Å  Met103:C----C:Rimonabant to 5.23 Å  Phe102:C----Cl:Rimonabant to 4.89 Å  Phe170:C----Cl:Rimonabant to 5.07 Å  Val196:C----Cl:Rimonabant to 4.33 Å  Met103:C----Cl:Rimonabant to 5.02 Å  Val196:C----C:Rimonabant to 4.51 Å  Val196:C----Cl:Rimonabant to 4.58 Å  Leu387:C----Cl:Rimonabant to 5.10 Å | Leu387:C----C:Rimonabant to 5.18 Å  Cys386:C----C:Rimonabant to 5.13 Å  Trp356:C----Cl:Rimonabant to 5.49 Å  Leu359:C----C:Rimonabant to 5.33 Å  Phe268:C----Cl:Rimonabant to 5.39 Å  Leu193:C----Cl:Rimonabant to 5.14 Å  **π-π interaction:**  Phe170:C----C:Rimonabant to 5.49 Å |
