# Supplementary figures and images for: Novel CB1 receptor antagonist BAR-1 modifies pancreatic islet function and clinical parameters in prediabetic and diabetic mice
Source: Nutr Diabetes. 2020 Mar 4;10:7. doi: 10.1038/s41387-020-0110-0 (PMC7055595; doi:10.1038/s41387-020-0110-0)

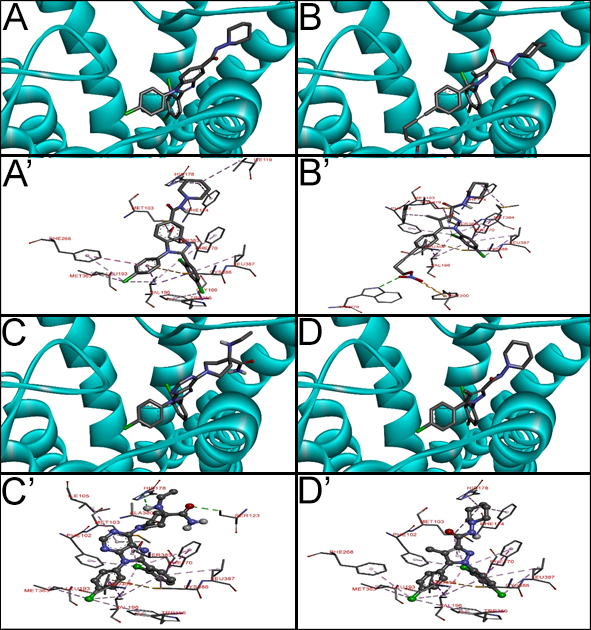

Supplement: Supplementary file 3 — Suplemental material - Figure A [file 41387_2020_110_MOESM3_ESM.tif]

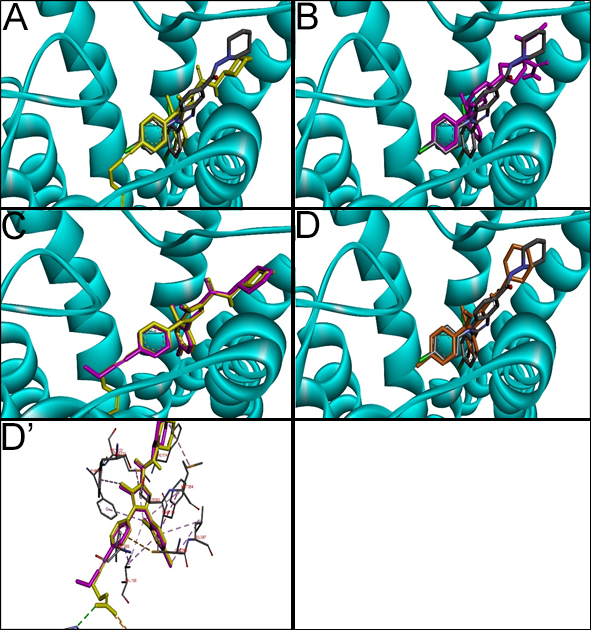

Supplement: Supplementary file 4 — Suplemental material - Figure B [file 41387_2020_110_MOESM4_ESM.tif]
